# Pan-London tuberculosis services: a service evaluation

**DOI:** 10.1186/1472-6963-12-203

**Published:** 2012-07-18

**Authors:** Ruth Belling, Susan McLaren, Markella Boudioni, Leslie Woods

**Affiliations:** 1 Institute for Leadership and Service Improvement, Faculty of Health and Social Care, London South Bank University, 103 Borough Rd, London, UK

## Abstract

**Background:**

London has the largest proportion of tuberculosis (TB) cases of any western European capital, with almost half of new cases drug-resistant. Prevalence varies considerably between and within boroughs with research suggesting inadequate control of TB transmission in London. Economic pressures may exacerbate the already considerable challenges for service organisation and delivery within this context. This paper presents selected findings from an evaluation of London’s TB services’ organisation, delivery, professional workforce and skill mix, intended to support development of a strategic framework for a pan-London TB service. These may also interest health service professionals and managers in TB services in the UK, other European cities and countries and in services currently delivered by multiple providers operating independently.

**Methods:**

Objectives were: 1) To establish how London’s TB services are structured and delivered in relation to leadership, management, organisation and delivery, coordination, staffing and support; 2) To identify tools/models for calculating skill mix as a basis for identifying skill mix requirements in delivering TB services across London; 3) To inform a strategic framework for the delivery of a pan-London TB service, which may be applicable to other European cities. The multi-method service audit evaluation comprised documentary analysis, semi-structured interviews with TB service users (n = 10), lead TB health professionals and managers (n = 13) representing London’s five sectors and focus groups with TB nurses (n = 8) and non-London network professionals (n = 2).

**Results:**

Findings showed TB services to be mainly hospital-based, with fewer community-based services. Documentary analysis and professionals’ interviews suggested difficulties with early access to services, low suspicion index amongst some GPs and restricted referral routes. Interviews indicated lack of managed accommodation for difficult to treat patients, professional workforce shortages, a need for strategic leadership, nurse-led clinics and structured career paths for TB nurses and few social care/outreach workers to support patients with complex needs.

**Conclusions:**

This paper has identified key issues relating to London’s TB services’ organisation, delivery, professional workforce and skill mix. The majority of these present challenges which need to be addressed as part of the future development of a strategic framework for a pan-London TB service. More consistent strategic planning/co-ordination and sharing of best practice is needed, together with a review of pan-London TB workforce development strategy, encompassing changing professional roles, skills development needs and patient pathways. These findings may be relevant with the development of TB services in other European cities.

## Background

London has the largest proportion of tuberculosis (TB) cases of any western European capital [1], accounting for 39 % of all new TB cases in the UK [2], compared with 15 % in the late 1980s [3]. In 2007, 3313 provisional new cases were identified in London [3]. Overall, rising incidence statistics suggest inadequate control of TB transmission in London. Undiagnosed and untreated, the death rate from TB is as high as 1 in 5. Case distribution within London varies considerably across geographic areas, ranging from 3 per 100,000 in Bromley to 89 per 100,000 in Newham [4]. In addition the nature of TB in London continues to change: 42 % of new cases reported from 1995 to 2005 were resistant to one or more drugs; risk groups include new UK entrants, the homeless and people with HIV [5]. Most TB cases are treated on an outpatient basis with the average cost per case to treat estimated at £10,344 [6]. However, drug resistant cases are estimated to cost some £60,000 [7] and multi drug resistant cases requiring hospitalisation can cost up to £250,000 [6]. Concern about failure to complete treatment regimes (particularly in socially excluded groups such as the homeless, intravenous drug users and prisoners) suggests that new models of outreach may be required.

National guidance on the management of TB [4], consistent with World Health Organisation guidance, forms the basis of best practice TB care in the United Kingdom [8,9]. However, by mid-2007 it was unclear whether this extensive guidance had been distilled into integrated care pathways for different types of clients within London. At the time of conducting this evaluation, TB services were currently provided across London by individual NHS Trusts and partner hospitals. Treatment was provided at 30 centres across the city and patients could attend clinics outside their borough of residence. Few had the critical mass of patients or staff to develop specialist services. The London Assembly recommended one whole time equivalent nurse/Health Visitor (with ‘full’ administrative support) for every 40 TB notifications [10]. In practice, this varied from 1:100 in Barking and Dagenham to 1:23 in Barts and the London NHS Trust and Tower Hamlets [6]. No equivalent recommendations existed for provision of medical staff, outreach or social care workers. Overall, these statistics suggested considerable challenges existed for TB service delivery in London; hence NHS London made TB a healthcare priority for 2007–2009 for all Primary Care Trusts (PCTs) [2].

Five multidisciplinary networks of TB services and professionals (geographically covering the historical boundaries of five former Strategic Health Authorities), three in the north of the city (North East (NE), North West (NW), North Central (NC)), two covering the south (South East (SE), South West (SW)), continue to coordinate the development of local services, each network supported by both managerial and TB health professional medical and nursing leads. A single ‘Stopping TB in London’ group developed to oversee the management of the drug resistant outbreak in North/North East London, steer the pilot on early identification of TB in high risk groups, as well as maintaining an overview of action across London to tackle TB. Considerable challenges exist for service organisation, delivery by healthcare professionals operating within this complex and varied context. This paper reports selected findings from a pan-London TB service evaluation in relation to service organisation, delivery and workforce issues and is intended primarily to support development of a strategic framework for a pan-London TB service. However, these findings should also interest health service professionals in UK TB services, other European cities and countries, as well as other health service managers considering skill mix issues. In addition, the evaluation approach based on combined methods may be relevant in other health services delivered by multiple providers, particularly those taking a strategic approach to workforce skill mix or needing to commission or review services.

### Objectives

1) To establish how pan-London TB services are structured and delivered in relation to leadership, management, organisation and delivery, coordination, staffing and support; 2) To identify tools/models for calculating skill mix as a basis for identifying skill mix requirements in delivering TB services across London; 3) To make recommendations which could inform a strategic framework for the delivery of a London TB service.

## Methods

This service audit-evaluation combined predominately qualitative methods: documentary analysis, semi-structured interviews and focus groups. Interviews with TB service users were also conducted; details of the purposive sampling strategy used and findings from TB service users are reported in a separate paper [11].

### Documentary analysis

A non-systematic audit evaluation of documentary evidence was conducted. Inclusion criteria: 1) public domain documents, published between January 1995–2007, 2) with specific reference to TB service organisation or delivery in or across London. No exclusion criteria were set regarding document type or study design. Search strategy focused on: electronic databases including Medline, CINAHL, Internurse; websites of global organisations, e.g. World Health Organisation; TB specific or related groups, e.g. Stopping TB London in group, TB Nurses’ Forum, British Thoracic Society. In addition, London sector TB networks provided service audit and mapping data, TB reports and epidemiological data. Search terms included: ‘tuberculosis’, ‘TB’ and ‘London’. The initial search generated 86 document references of which 17 met the inclusion criteria. Due to the variable nature, structure and content of the included documents, analysis of their content using the thematic approach adopted for interviews was not possible. All documentary sources were initially categorised and analysed according to key project objectives utilising structured headings of leadership, management, organisation and delivery, coordination, staffing support and skill mix to form a structured framework. It was then possible to link this with emergent themes arising from the thematic analysis of focus groups and semi-structured interviews. For example, service structure and delivery; key challenges for delivery; networking/co-ordination; skill mix. Findings from the documentary analysis and interviews have been integrated in the results section of this paper.

### Semi-structured interviews and focus groups

To obtain a wide range of perspectives on how London’s TB services should be structured and organised, views were sought from the following key groups of health professionals and managers a) professional TB leads and managers representative of the five London sectors (n = 15); b) TB specialist nurses working in London (self-selected through open invitation via TB Nurses’ Forum); c) non-London network TB professionals (selected using snowball sampling methods, including listings in TB specialist group publications and referral by London sector professionals). Interviews with a purposive sample of TB service users (n = 10) were also conducted and are described in more detail a separate paper [11].

A total of 23 health professionals took part in the interviews and focus groups, which were of 30–60 minutes duration. Semi-structured interviews were conducted with 13 lead TB doctors, nurses and managers representative of the five London sectors. Two focus groups, one with eight TB nurses and one with two non-London network TB/respiratory physicians were also completed. Interviews with 11 of the health professionals were tape recorded; handwritten notes were taken during two interviews due to extraneous noise from the interview surroundings. Both focus groups were tape recorded and summary notes also made on flip charts. Following transcription of recordings, all qualitative data was imported to the QSR Nud*ist v.6 software package and systematically analysed following a thematic approach, coding data into units of meaning, building these into categories and themes based on similarities and differences [12]. Eight main themes and 39 sub-themes were identified. Of these, four main themes (service structure and delivery; key challenges for delivery; networking/co-ordination; skill mix) and selected sub-themes impacting on service organisation, delivery, workforce and skill mix are presented below using anonymised, illustrative extracts, with all respondents randomly assigned a unique three-digit code.

## Results

### Service organisation and delivery

Documentary analysis of sector TB service audits, supplemented by health professionals’ direct knowledge, identified 30 TB treatment centres in London, predominantly hospital-based, funded by either acute or primary care trusts, allowing in-patient care, access to specialised facilities and diagnostic services, with the majority of TB cases treated in outpatient clinics during regular weekday office hours (Mon-Fri, 9 am-5 pm). A minority of hospital-based services offered community-based clinics; fewer services were principally community-based. These community-based services were set up in response to local needs, particularly within North London sectors where the proportion of drug-resistant TB cases had been highest: for example, in NE sector, access to services was facilitated by self- referral with some walk in clinics.

In terms of sector specific models for service delivery, three sectors (NE, NC, SE) could be broadly classified as central with satellites, although some variability was noted. The inception of a single provider, hub and spoke variant to this model, in which a central employing Trust (NC) provided services to six others, was a relatively new development driven by rising TB rates, serious outbreaks of drug resistant disease, needs to rationalise service level agreements, flexible deployment and co-ordination of services. One sector service was specialist community based (NW) and another specialist hospital based (SW). Within sector models a number of examples of shared service delivery were evident, one characterised by joint working between TB specialist nurse and school nurse, to the benefit of newly diagnosed children and parents. Other interesting pilot service innovations included the inception of a TB specialist nurse post within a prison health service where a focus was placed on the development of a TB protocol and policy [13]. Exemplars of working across organisations included external liaison with asylum and refugee voluntary groups within one sector.

Based on documentary analysis, pan-London TB services were audited in relation to interim progress with the achievement of nine draft quality targets listed below, derived and developed from ‘Stopping TB in London’ [6]. Indicators enable services to measure progress against targets, creating benchmarks for future pan-London service comparisons which are integral to a performance management framework for TB services:

1. To prevent TB in babies and young children. (Meeting incidence related targets for BCG provision).

2. To provide early access to specialist TB services. (Patients seen by specialist TB service within 2 weeks of GP referral).

3/4 To ensure early diagnosis. (Processing of TB laboratory samples using liquid culture technology and results on sputum smears to be available within one working day of the sample reaching the laboratory).

5. To identify patients with complex needs. (A risk assessment as defined by National Surveillance Standards conducted to identify those at risk of treatment non-completion.)

6. To achieve a minimum of 85 % treatment completion rates.

7. To prevent further infection through contact tracing. (Contacts traced and screened arr. NICE guidelines.) [4]

8. To provide a TB specialist nursing workforce target of 1:40 notifications and full clinic administrative support in place.

9. To offer an HIV test to anyone over the age of 16 with diagnosed TB.

Service mapping of data based on annual reports for 2006 from three sectors (NW, NE, SE), the ‘Stopping TB in London Group Report’ [6] (for NC and SW) and results documented from other local sector audits (internal and external) against these draft targets, provided an interim picture of sector progress. Overall, sectors performed best against four of the nine draft targets, fully or partly meeting targets concerning prompt diagnosis by laboratories, 85 % minimum treatment completion and prevention of further infection through contact screening. Though sectors performed well in relation to early diagnosis targets however, these reflected performance and turnaround times of laboratories involved in processing diagnostic tests. Specialist TB nursing workforce targets were based on British Thoracic Society (BTS) guidelines. In NE and NW sectors, just over half the providers and clinics had fully or partly met this target. Audit data for specialist TB nurses to TB notifications ranged from 1:20 to 1:100. However, there was no clear evidence overall that nursing and administrative staffing levels across London had improved on figures reported by the BTS in 2002 [14].

Health professionals’ interviews and focus groups highlighted additional important service issues not currently addressed by sector targets, but perceived as negatively impacting early diagnosis and disease transmission control, most notably: restricted referral routes, low index of suspicion particularly amongst GPs and a lack of TB awareness amongst patients and primary care staff.

### Access and referrals

Both medical and non-medical health professionals indicated a wide variety of access routes into the TB service:

"“Mainly through A&E, GP, occasionally self-referral… through contact tracing. Sometimes through other organisations, e.g. drug teams at [named], but not that often. Certainly, in the south of sector [named], there is a large homeless population and they have quite a good relationship with hostels and they would be referred in through their key worker. Also, the mobile x-ray unit.” (001)"

For the majority, access was via GP or clinic referral, although some hospitals had a walk-in facility allowing patients to be seen urgently. Many professionals felt services were able to better the national target for seeing patients within two weeks of presentation:

"“There is a target for having patients seen in two weeks, but majority of clinics try to see patients within 48 hours, which is very good.” (012)"

Some focus group nurses reported that the difficulty was not in seeing patients within two weeks, but starting them on treatment within that period and many were keen to develop faster access routes, for example through an on-site nurse three days per week, with referrers contacting the nurse to enable urgent cases to see a doctor more quickly.

Other participants reported referral processes differing widely across even localised services, with some restricting access for seeing suspected TB cases to GP referral only, despite GP shortages and registration requirements, arguably making it difficult for people within some of the highest risk groups of contracting TB to actually register with a GP. In such cases, access to TB services was reported to occur by presentation through emergency routes such as hospital casualty services:

"“Quite a few people come in through A&E, usually the ones who haven’t got GPs.” (005)"

Issues were also raised regarding initiatives aimed at promoting patient choice which were perceived as conflicting with professionals’ attempts to meet TB targets and problems obtaining appropriate referrals due to a low index of TB suspicion particularly amongst GPs.

"“But getting referrals in the first place is a problem. TB was misdiagnosed or not suspected at all, so [service named] is the only [service] that’s actively reaching out to GPs, trying to raise their index of suspicion for TB. Even though TB rates are high in London, it’s not so common that a GP will recognise it.” (004)"

Countering low index of suspicion, promoting screening and referral in general practices represents a considerable challenge. The documentary analysis identified one randomised controlled clinical trial which evaluated an educational outreach programme as a way of promoting TB screening in general practices in Hackney [15]. Findings showed that in intervention practices 57 % of patients attending a health check were screened for TB (verbal assessment proceeding to Heaf test where appropriate), compared to 0.4 % in control practices. Results also demonstrated a greater increase in intervention practices in diagnosis of latent TB. Although limited sensitivity of the Heaf test and contextual study limitations were acknowledged, conclusions were that educational outreach interventions improved active and latent TB identification.

### Network coordination

Focus groups and interviews suggested networks, though at different stages of development, operated well within sectors:

"“It tends to work better within each sector or within hospitals… We do have good relationships and we can ring somebody in other sectors and talk about particular patient issues. But we’re not really cohesive.” (001)"

Networking between sectors, however, was less well established, but seen as beneficial, with logistical issues cited as a reason why multiple sector networks had been difficult to co-ordinate.

"“Good at working within and sharing with each other, but not so good at networking out.” (012)"

"“Networking across the whole of London has been difficult. It’s the size of London and also the perceptions of what’s important.” (008)"

Another obstacle to networking between sectors, was the lack of funding for a formal TB Network Manager/Co-ordinator in one sector, which had meant limited representation for this sector, a situation forthcoming performance management requirements were expected to exacerbate.

"“In the south, PCTs have not always provided support and money to the networks. I’ve never understood why there was a problem. Overall, individual hospitals work well. But co-ordination across that sector means there’s an incredibly variable delivery of TB services, number of doctor sessions per patient etc.” (002)"

"“I think the funding would have been more easily found for a network manager, had [named sector] had the numbers of cases that would have kept [named sector] really busy. With performance management coming in, that really can’t be done without a network manager. Somehow the PCTs are going to have to get together and decide between them how to fund one.” (005)"

Professional bodies and cross sector professionals’ groups provided valuable resources in combating professional isolation, notably the London TB Nurses’ Forum. However, participants reported this group was no longer meeting regularly and lacked a longer term element of continuity that could be provided by a fixed meeting chair. Interviews also suggested that a single pan-London network would be welcomed amidst calls for a stronger and more strategic overview of TB services:

"“The networks work well. What is missing is someone managing or overseeing the whole picture.” (003)"

"“I don’t see any strategic planning around the networks… strategic thinking tends to be local rather than London wide.” (006)"

Such a network was perceived as enabling more consistent planning, agreement, coordination and implementation of TB services across London, as well as providing an appropriate forum for broader discussion of legislative, ethical and research issues impacting across services. Focus group non-network professionals said that strong professional TB networks were needed to support services, in addition to appropriate funding and infrastructure. A national network also needed to develop, which could in turn contribute internationally, with representation from service users as well as health professionals.

### Leadership and management

Focus groups and interviews suggested there were some strong sector leaders, driving London’s TB services to meet local needs. However, a perceived lack of leadership beyond individual sector boundaries and at more strategic levels, suggests major organisational challenges for London’s TB services, apparent in the concern expressed by all professional groups that TB needed greater prioritization at an organisational level, particularly in acute Trusts where some TB teams perceived tensions and lack of support in carrying out the public health and outreach aspects of their roles:

"“There are few strong leaders and little being done to support those strong leaders. So those who do want to go out there and take the risk, both at local and sector level, taking risks is not supported. It’s seen as a maverick thing to do and so they don’t feel confident about going out there and they become disenfranchised. So, leadership’s a clear issue.” (006)"

Within the TB workforce there was a clear demand for more nursing leaders with the skills and experience to increase and develop nurse-led services. In addition, one sector was operating without a formal lead TB nurse, raising concerns that senior nurses were not being empowered locally. For the highest nursing bands there was an urgent development need to enable more nurse-led clinics to operate, freeing up physicians’ time as well as providing a more patient focused service. In the longer term, there was a need to train and develop junior nurses in management skills, thus ensuring a suitable cadre of future nursing managers and leaders:

"“Need to start management process and experience by making nurses have management responsibilities at Band 6.” (003)"

There was a consensus in every sector, amongst every professional group interviewed, that nurses could and should play a vital role in leading patient care, not only in the management of less medically complex patients, but also in providing after care and organising and running clinics. However, the proportion and state of development of nurse-led services and clinics differed widely between and within sectors:

"“Most services are nurse-led, but some still consultant based.” (006)"

"“There are very few nurse-led clinics.” (009)"

Many nurses found it difficult to articulate and define the meaning and constitution of ‘nurse-led’ service. Wide practice variations may contribute to the lack of a shared view. Some nurse-led services were restricted to operating more routine clinics where the administrative workload is arguably more intense but the majority of cases less medically complex:

"“Contact screening clinics are nurse led and follow up clinics too.” (007)"

Some had a far more extensive and proactive remit, taking full charge of tests and assessments before a diagnosis is made. Key to the success of such proactivity was the full support of medical consultant colleagues:

"“We do all the assessments and tests, whatever happens. Then, with the results, we go to the consultants and say this person might have TB. So all the services in [named area] are nurse led, but with a very, very supportive consultant.” (008)"

Other services were almost completely nurse-driven, maximising the contributions of the whole TB team, enabling physicians to see patients when their input was vital and allowing nurses to take on a wider variety of tasks, increasing responsiveness and benefits to patients. Nurses’ focus group respondents said nurses were best placed to “deliver the whole package”, suggesting that ideally the patient’s first meeting should be with a consultant. Thereafter, nurses would co-ordinate the rest of patient care with appropriate consultant input.

### Inadequate staffing and support

Inadequate staffing levels amongst physicians, TB nurses and administrative staff were consistently highlighted by all professional groups as a major challenge for TB service delivery. In some sectors, there were insufficient numbers of experienced TB physicians and not enough physician time allocated to TB. For some, the TB component of their work was not contractually and explicitly specified:

"“If you look at any TB doctor, it’s not really written down in their job plan. For instance, it doesn’t say anywhere, you will do tuberculosis. It says you will be lead for TB… And that’s quite different from TB nurses or TB social workers, where it actually says that’s what they do.” (011)"

"“The only problem is that they do a lot of other things as well. None of our clinics has enough workload to support a full time TB physician, but they don’t have enough time to do TB. Most of them do more than their contract time in TB. We’re very lucky.” (007)"

Non-network professionals said that with changes to doctors’ education, some junior doctors now needed greater support in taking on medical tasks that might otherwise have freed up more experienced physicians’ time. Also, that time spent on education was not always being accounted for.

Most sectors had highly motivated and committed physicians, but one sector had experienced difficulties, which highlighted the scarcity of experienced TB doctors and the negative impact on service delivery of the loss of even one individual.

"“Keeping clinicians interested in TB and having Trusts recognise they need sessions devoted to TB care. They don’t have enough sessions at clinics. One of the most experienced clinicians has resigned and the locum at present doesn’t have the same interest in TB. To lose the place and have someone without that interest is a serious loss. So we’re really teetering on the brink there. There are significant challenges in keeping a status quo let alone developing it.” (012)"

In addition to the service delivery challenges posed by the shortage of TB physicians, specialist TB nurses consistently reported high workloads not adequately reflected in the British Thoracic Society’s guideline staffing ratio of one specialist TB nurse to 40 TB notifications, due to time spent with increasing numbers of socially and medically complex TB index cases, contact screening and patients on chemoprophylaxis.

"“You can have ten patients, but they may be very complex. Or you have forty patients, but you’ve got all the contact screening to do. You might only end up with another one patient, but you still have to do that work. And you might have an outbreak in a school. It doesn’t take account of clinics. Only the index case.” (001)"

"“I try to include a caseload as well, but Trusts are only interested in notifications per nurse. I don’t think the 1:40 will change. Ideally, I think it should be less than that. It’s up to those clinics and nurse managers to advocate for themselves and make a case for why they need another nurse, for example. I also look at full-time equivalents, rather than the physical number of nurses or it looks like there are lots of nurses when there aren’t. It would help to look at treatment completion by clinic, for example, because people don’t only go to the clinic in their [local area], so by [local area] doesn’t give an accurate reflection.” (004)"

Interviews also highlighted problems of retention, frozen posts and redundancies which constituted a risk both for maintaining current levels of service delivery as well as future workforce levels and development:

"“Nurses need help with recruitment and retention, resource issues, training and planning services.” (009)"

"“And in some areas of London, where nurses are being told to stop being specialist TB nurses and start being more general respiratory nurses, say, or even the post has gone, I think this is crazy. I think one has to think very carefully about the utilisation and professional development of nursing staff, because they’re such a strength.” (011)"

In some acute settings nursing posts were already split between TB and more general respiratory work:

"“When there are problems the respiratory side gets dropped. It has to. That’s a large issue for this trust because we’ve had four incidents where we’ve had to screen a lot of people, so it has involved a lot of work for everybody… But that’s not ideal because you’ve always then got this backlog of patients in other areas.” (008)"

"“If I was setting up from scratch I’d try to make sure nurses were either TB or respiratory.” (005)"

Across London as a whole, concerns were raised about sustaining an adequate pool of trained TB nurses:

“In London, you’re not getting enough nurses coming up through the ranks to be able to maintain the service at the same level. We shouldn’t be battling to get people upgraded, purely because you took them on as a training post.” (008)

### Social care/outreach workers

In addition to the perceived lack of TB physicians and nurses, professional staff identified social care/outreach workers as potentially supporting TB service delivery. At the time of this evaluation, the development of social outreach worker posts within TB services was still relatively new, although documentary analysis confirmed that such posts were represented in all North London sectors where the proportion of medically and socially complex, multi-drug resistant cases are highest and two sectors (NC, NE) were employing social care and outreach workers as part of their TB service teams:

"“They also have an outreach worker, though that post is vacant at the moment. [named area] and one other have outreach workers, though the worker only works one day a week and for another clinic too. I think outreach workers are very important.” (004)"

Documentary analysis highlighted one process evaluation of a TB link worker role in North London [16], part of a three year pilot to develop a social outreach care model to meet the health and social care needs of homeless, prison and drug and alcohol-using TB patients, which found the majority of patients’ needs were housing or welfare claims related. Agencies involved reported 17 separate benefits of the link worker post, including additional time, intensive support, information sharing and disease awareness raising. Community education and resources were recommended to support link workers where nurses were unable to perform educational outreach and in this instance a nursing post was subsequently reconfigured to provide continued funding.

Echoing these findings, both medical and non-medical TB professionals reported social care and outreach staff as being of particular assistance in areas with high proportions of homeless patients, immigrants, refugees, asylum seekers, prison populations and patients with drug abuse, freeing up nursing staff from some of the most labour-intensive activities, while supporting difficult to reach patients in taking prescribed medication and staying on treatment:

"“…we’ve increased our social care input, because we’ve seen that as a really important component of what we need to provide here. We have a lot of homeless, a lot of people connected to prisons, a lot of people who are drug users or linked to former drug use. And we have a large immigrant population who are asylum seekers and also a lot of mental health problems.” (011)"

"“For instance, our outreach worker is not from a nursing background, but she can phone up housing, social services, psychiatric services, make sure patients get their money and don’t spend it all on crack or heroin.” (002)"

Directly Observed Therapy and dealing with patients’ social care needs were the most frequently mentioned tasks where non-nurses might appropriately be employed and many nurses taking part in the focus groups were keen to have more social care/outreach/case workers attached to their teams. Benefits identified included relieving workload pressures on over-stretched nurses, providing a non-nursing view for patients, visiting patients at home, helping TB teams better understand patients and ability to access hard to reach communities.

Others, however, were less certain about the benefits:

"“There have been moves to have more outreach workers, but the outreach workers come from an ethnic group and one ethnic group might not like another ethnic group. A lot of my patients express a wish to be seen by someone who’s been born and brought up here, because they think we will be fairer and won’t use the ethnic card against them.” (010)"

Some sectors which have seen social care and outreach workers as a valuable resource, had been unable to obtain funding, particularly in areas where patients are treated in clinics outside their residential area. In one sector, funding from a vacant nursing post was diverted. Others had been forced to retrieve income from hospitals and reinvest it in TB services to try to mainstream posts.

A variety of designations were clearly in use, including outreach worker, support worker, social care worker, social worker, case worker, link workers and advocates. Some titles appeared to be used interchangeably and it was not clear whether post holders were actually performing similar tasks, suggesting clarity is needed to differentiate between the roles, responsibilities and activities of this emerging workforce group, in considering London’s overall TB service skill mix.

### Skill mix requirements

The Stopping TB in London Group Report [6] identified five areas of skills needed within TB teams:

1. Clinical skills: assessment, nursing care, planning and research

2. Social health skills: advocacy, housing, dependency/addiction, immigration, treatment support

3. Management skills: leadership, staff management, budgetary

4. Educational skills: training, health promotion

5. Administrative skills: office management, data collection, audit collection.

Apart from information cited above, documentary analysis identified no TB service-specific tools, assessment instruments or models for calculating or determining skill mix in use across London. However, a more generic approach to skill mix review which has involved the development of a decision support tool for managers, could be a helpful starting point in formulating an approach for local TB services, as could international reviews on the evidence for skill mix [17,18].

Documented approaches to professional skill mix did not extend beyond specialist nursing and administrative support ratio guidelines provided by the British Thoracic Society [10]. Detailed and comprehensive documentary information relating to multidisciplinary working in TB teams (characterized by generic and specialist skills) were not identified. However, sector specific service models were characterized by variable professional skill mix.

Interviews and focus groups revealed that healthcare professionals relied on individual approaches to estimating staffing levels, in attempts to develop realistic formulas adapted to their particular sector contexts.

"“I try to include a caseload as well, but PCTs are only interested in notifications per nurse. I don’t think the 1:40 will change. Ideally, I think it should be less than that. It’s up to those clinics and nurse managers to advocate for themselves and make a case for why they need another nurse, for example. I also look at full-time equivalents, rather than the physical number of nurses or it looks like there are lots of nurses when there aren’t. It would help to look at treatment completion by clinic, for example, because people don’t only go to the clinic in their [local area], so by [local area] doesn’t give an accurate reflection.” (004)"

No consensus was reached by TB professionals interviewed or in focus groups as to the best ways to determine appropriate skill mix. Nevertheless, TB professionals, particularly those working in the highest TB incidence sectors, were very clear that however skill mix was considered, provision was and should be based on a combination of local service needs and specific outcomes.

"“It’s determined by local need. We have people working with HIV, alcohol, specialist addiction unit, because that’s what we need. It’s silly to think of huge teams where every possibility is covered. It’s much more sensible to have people you can get advice from on how to approach a particular problem, by email for example. Once you get people in too large groups it begins to fragment. Better to have people in smaller groups that work efficiently.” (002)"

"“I think it’s a combination of what’s locally felt to be appropriate, some strategic planning at a PCT level and you have what you have. If you only have one nurse, that’s your skill mix and that’s it. If you have supportive management and a flexible structure, you can do all sorts of things. But here, services are beginning to be determined by need and informed commissioning, so we should be commissioning services that get us to the outcomes, rather than saying we need a minimum number of x staff.” (006)"

Focus group nurses identified a minimum skill set for TB services based on professional status as comprising one consultant and one TB nurse with appropriate administrative support. An ‘ideal’ service was described as the above minimum plus a psychologist, social worker, linked to pharmacy, infectious disease and microbiology facilities.

## Discussion

Key objectives of this evaluation were to identify how London’s TB services were structured, organised and delivered, and to identify tools/models for the determination of skill mix requirements for TB services. The use of combined methods has yielded some important and interesting findings which can be used as a baseline to inform future development of a strategic framework for pan-London TB service delivery. Furthermore, little has been previously published which represents a qualitative exploration of the views and experiences of TB professionals which are key to informing future service delivery. Lack of timely and consistent sector-wide information hindered the intention to produce a comprehensive picture of services: this was partly due to evolving performance targets and criteria, but also varying resource levels and mechanisms within sectors. Without these it is difficult to establish and maintain a clear and vital overview of service delivery and performance on which to base future strategic planning. This, together with resourcing implications, therefore need to be addressed at a pan-London level and prioritised to ensure a consistent, accurate and effective information database to underpin future service planning.

### Service access and referral

Findings identified diverse modes of access to TB services, encompassing GPs, contract tracing, via key workers from social services, self-referrals, provision of hospital walk in centres and mobile x ray unit screening. Also highlighted by this evaluation were significant issues concerning variable and inflexible access to TB services, for example solely GP referral in some sectors, despite GP shortages and registration requirements, contributing to the utilisation of potentially more costly access routes via hospital accident and emergency services. Flexibility of service access more generally has been an issue in other major cities, for example, New York, where opening chest centres outside normal business hours has been piloted [19,20]. It was encouraging to find that in some London sector clinics the target of providing early access to specialist TB services (6) could be lowered from 2 weeks to 48 hours. However, professional staff also reported instances of a low index of suspicion amongst GPs as a barrier to referral, a problem also identified by service users in a separate component of this evaluation [11]. Raising GP awareness through educational updates together with utilisation of educational outreach programmes in GP practice settings [15] can offer a way forward.

### Co-ordination of sector TB services

London’s five TB sector networks were perceived to be operating well, within sectors, with multidisciplinary meetings largely well attended in each. Extending networks to create better links between sectors, was welcomed by health professionals as a means of enabling greater consistency in service planning, coordination and implementation. Its achievement, however, is hindered by logistical difficulties and lack of prioritisation, suggesting more centralised support in the form of a cross-sector manager/co-ordinator could be required to increase the effectiveness of cross-sector networking.

Different service models were identified in operation within London sectors, which had evolved to meet local needs. An evaluation of the single employer provider of TB services to other Trusts (NC), which carries the potential for flexible deployment of staff, rationalisation of service level agreements and improvements for service co-ordination, would be helpful. Although no consensus was agreed about an optimal pan London service delivery model, many health professionals welcomed the concept of a single pan London network aligned to strong strategic leadership as a way of achieving more effective service co-ordination and delivery. Future strategic planning could benefit from reviewing service models in operation in other cities of high TB incidence, where a measure of control has been achieved. For example, the inception of a single organisation with responsibility for pan city TB control, managing multiple providers, has been deployed successfully in New York [21].

### Leadership and management

Findings highlighted considerable challenges for leadership at both strategic and operational levels within London’s TB workforce. There was a clear demand for more nursing leaders with both skills and experience to develop and increase nurse-led services, with perceived benefits in freeing up doctors’ time as well as enhancing patient centred care. Despite little consensus over definition and extent of ‘nurse-led’ services, there was unequivocal support for the view that these would only be successful in the context of mutually supportive relationships with medical colleagues. Allied to this was the perceived lack of structured career paths for TB nurses and a need to create greater opportunities for Band 5/6 nurses to develop leadership skills earlier in their nursing careers. This suggests stronger links may be necessary with both professional regulation [22] and national policy underpinning modernisation of both nursing and medical careers [23-25]. Investment in leadership and management training which are informed by the Leadership Qualities Framework [26] is recommended.

### Workloads, role development and skill-mix

Inadequate staffing levels amongst physicians, TB nurses and administrative staff, continue to constitute a major challenge for TB service delivery. Many areas of healthcare have sought to maximise clinicians’ time through increased development of nurse-led services [25]. While this study’s findings indicate perceived benefits and support for such developments within London’s TB services, serious challenges exist for successful implementation and sustainability due to inadequate levels of specialist TB nurses, lack of nursing leadership, leadership development opportunities and nurses’ increasingly high workloads due to increasing numbers of socially and medically complex TB index cases, contact screening and patients on chemoprophylaxis.

This evaluation found no clear evidence of improvement in nursing and administrative staffing levels across London since those reported by the British Thoracic Survey [12]. It has, however, identified several key contributory factors to the persistent problem of securing TB nursing workforce levels: recruitment and retention difficulties, redundancies, frozen posts, split posts and lack of structured career pathways linked to both professional regulation [22] and policy agendas for modernising nursing and medical careers [23-25].

While the issue of high workloads could be addressed as part of an overall workforce skill mix review by applying weightings for complex patients to case loads and by development of link/social support worker roles to free up nursing time, one potential solution has been to develop social care/outreach posts in response to increasingly socially complex needs of those most at risk of not completing treatment. This evaluation found evidence of several sectors, particularly those with high proportions of such cases, currently employing social care/outreach workers as part of their multidisciplinary TB services and in common with Craig et al [16], experiencing valuable benefits for both staff and service users. The plethora of designations, roles, responsibilities and activities of this emerging workforce group, however, suggests an unmet need for role clarification and the development of sustainable funding models to ensure the inclusion of relevant skills within London’s overall multidisciplinary TB service skill mix. It is therefore recommended that representatives of this group should be included in any future review of skill mix, workloads and role development.

A pan-London workforce skill mix and role development review may also be usefully informed by consideration of future Advanced Nurse Practitioner and Assistant Nurse Practitioner roles and by further investigation of how multidisciplinary TB teams function in relation to the acquisition of selected generic skills, a model operating in community mental health teams in the UK, designed to maximize efficiency and effectiveness. Findings of this study also identified that health professionals’ perceptions and understanding of skill mix varied; a finding consistent with those of Buchan and O’May [17]. Although no TB services specific approaches to skill mix determination were identified, a more generic approach offers a way forward, which views skill-mix as context specific and underpinned by realities of organisational change [18]. Since it is not specific to TB, this approach may therefore also benefit other health service professionals and managers considering skill mix initiatives within their organisations.

Although the scope of this evaluation was TB services, structures and organisation within London, TB is nevertheless a global problem, with multidrug resistant outbreaks affecting comparable sized cities with relatively well-developed healthcare systems [8]. With the exception of one comparative study focusing on Osaka City, Japan and London [27], no published documentary evidence was found comparing London’s TB service models and infrastructure with those of similar urban contexts. By analysing approaches which have proved effective in other major cities, such as New York [21], useful insights and options may be gained for future management and organisation of London’s TB services.

Further research is needed to evaluate alternative TB service models, such as the single hospital employer, community deployed nursing workforce model currently operating in one London sector and also the organisational structures and practices used effectively in cities such as New York and Amsterdam, which might be applied to other western cities. Future research should continue to evaluate multi-disciplinary skill mix configurations within TB services and leadership skills’ development among nurses to enable more flexible, needs-driven, locally delivered TB services for the future.

### Limitations

It is acknowledged that findings from focus groups and interviews were limited by only partial sector representation of medical staff and no direct participation by social care/outreach workers was included as part of the study design. Inclusion of the latter would have greatly enhanced the scope and interpretation of the qualitative findings. Documentary sources were also limited to those within the public domain and were therefore qualified by availability and timing.

## Conclusions and recommendations

This paper has identified key facets within London’s TB services’ organisation, delivery, professional workforce and skill mix. The majority of these present challenges which need to be addressed as part of the future development of a strategic framework for a pan-London TB service, encompassing changing professional roles, skills development needs and patient pathways. Recommendations are that (i) collection of sector performance data against TB targets is centrally co-ordinated and effectively resourced; (ii) low index of suspicion amongst GPs is addressed through in service education/updates and the wider inception of educational outreach based in GP practices is considered; (iii) leadership and management training for TB nurses is prioritised and further consideration is given to development of nurse led co-ordination of TB services; (iv) gaps in strategic leadership of TB services are addressed; (v) a skill mix review is conducted by a multi-disciplinary task group to consider both sector and pan London needs; (vi) a strategic review of alternative service models is conducted based on analysis of those reported in the international literature and those currently extant in London, including the single employer provider model. The evaluation approach used here, which utilised combined methods, may be relevant for other health services delivered by multiple providers in the UK or elsewhere, particularly those taking a strategic approach to workforce skill mix or needing to commission or review services.

## Competing interests

The authors declare that they have no competing interests.

## Authors’ contributions

RB participated in study design, document analysis, conducted and analysed staff interviews, drafted and revised manuscript. SM participated in study conception and design, document analysis, critical revision of manuscript for important intellectual content. MB participated in conducting and analysing service user interviews, critical revision of manuscript for important intellectual content. LW participated in study conception and design, document analysis and critical revision of manuscript. All authors approved final manuscript version.

## Pre-publication history

The pre-publication history for this paper can be accessed here:

http://www.biomedcentral.com/1472-6963/12/203/prepub
